# Effect of EGCG Intake on Kidney Function Chronic Kidney Disease: A Nonlinear Dose–Response Relationship and Health Impact Evidence From US Adults

**DOI:** 10.1002/fsn3.70997

**Published:** 2025-10-29

**Authors:** Xuetong Tang, Jiankui Guo, Wen Hu, Yuan Liu, Yunying Shi

**Affiliations:** ^1^ Department of Clinical Nutrition West China Hospital, Sichuan University Chengdu Sichuan China; ^2^ West China School of Public Health Sichuan University Chengdu Sichuan China; ^3^ Department of Nephrology West China Hospital, Sichuan University Chengdu Sichuan China

**Keywords:** chronic kidney disease (CKD), epigallocatechin gallate (EGCG), glomerular filtration rate (eGFR), NHANES, nonlinear dose–response relationship

## Abstract

Epigallocatechin gallate (EGCG), a major catechin in tea, has been linked to various health outcomes; however, its association with kidney function remains unclear. This cross‐sectional study included 5001 participants from the 2009–2018 National Health and Nutrition Examination Survey (NHANES). Daily EGCG intake was estimated from two 24‐h dietary recalls. Kidney function outcomes included chronic kidney disease (CKD), estimated glomerular filtration rate (eGFR), and urinary albumin‐to‐creatinine ratio (UACR). Multivariable logistic and linear regression models were used to assess associations, and restricted cubic spline (RCS) models were applied to examine potential nonlinear relationships. Per 100 mg/day higher EGCG, CKD odds did not change meaningfully: crude OR 1.03 (95% CI 0.93–1.15; *p* = 0.59) and adjusted OR 1.09 (0.97–1.23; *p* = 0.14). By contrast, EGCG was inversely related to eGFR: crude β −1.01 mL/min/1.73 m^2^ (95% CI −1.59 to −0.43; *p* = 0.0006) and adjusted *β* −0.86 (−1.23 to −0.49; *p* < 0.001). UACR showed no significant associations: crude *β* −13.09 (95% CI −89.26 to 63.08; *p* = 0.736) and adjusted *β* −22.49 (−103.87 to 58.90; *p* = 0.588). RCS indicated a nonlinear association for CKD (*p*‐overall = 0.021; *p*‐nonlinear = 0.010) with an inflection near 48.750 mg/day; for eGFR, the overall association was significant (*p*‐overall < 0.001) with a visually N‐shaped curve and a turning point near 52.707 mg/day, though nonlinearity was not significant (*p*‐nonlinear = 0.063). No overall or nonlinear relationships were observed for UACR (*p*‐overall = 0.764; *p*‐nonlinear = 0.752). Higher EGCG intake was consistently associated with lower eGFR, and a significant nonlinear association with CKD risk was identified, suggesting that the relationship between EGCG and kidney function may vary across intake levels. No association was observed with UACR.

## Graphical abstract

We analyzed NHANES 2009–2018 adults to relate continuous epigallocatechin gallate (EGCG) intake to chronic kidney disease (CKD), eGFR, and UACR using multivariable logistic/linear models and restricted cubic splines. EGCG was not associated with CKD in conventional models and showed no association with UACR, whereas each 100 mg/day higher EGCG was linked to a lower eGFR (adjusted *β* = −0.86 mL/min/1.73 m^2^; 95% CI –1.23 to −0.49). Spline analyses indicated a significant non‐linear association with CKD (*p*‐overall = 0.021; *p*‐nonlinear = 0.010) with an inflection near 48.750 mg/day, while eGFR's overall association remained strong without significant non‐linearity.

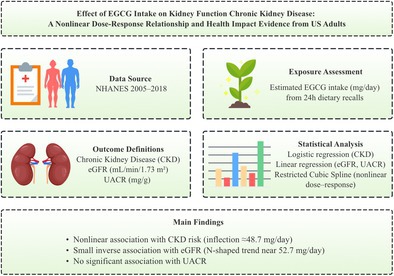

## 1 Introduction

Chronic kidney disease (CKD) is defined as a persistent abnormality in kidney structure or function, such as decreased estimated glomerular filtration rate (eGFR) or increased albuminuria, lasting for more than three months (Romagnani et al. 2017). Due to its high prevalence, debilitating nature, and significant burden on healthcare systems, CKD has emerged as a major global public health challenge. According to the 2017 Global Burden of Disease Study, CKD was responsible for 1.2 million deaths annually, reflecting a 41.5% increase since 1990, alongside a 29.3% rise in prevalence (Jha and Modi 2018; GBD Chronic Kidney Disease Collaboration 2020). CKD frequently progresses to end‐stage kidney disease (ESKD), a condition characterized by severely impaired kidney function that necessitates costly treatments such as dialysis or kidney transplantation, thereby exerting immense pressure on healthcare resources, particularly in low‐ and middle‐income countries (Rai et al. 2023; Xie et al. 2018). Moreover, CKD not only leads to a progressive decline in renal function but is also closely associated with other chronic diseases, such as cardiovascular diseases, which significantly elevate mortality risk and further strain public health systems (Herzog et al. 2011; Matsushita et al. 2010).

Tea, a widely consumed beverage in East Asia and worldwide, has garnered significant attention for its potential health benefits, especially in relation to chronic diseases (Khan and Mukhtar 2013). Green tea, in particular, is abundant in epigallocatechin gallate (EGCG), a potent antioxidant known for its anti‐inflammatory and lipid‐regulating properties (Vieira et al. 2019; Higdon and Frei 2003; Fujiki et al. 2023). The unique phenolic structure of EGCG allows it to neutralize free radicals and reduce oxidative stress, which may contribute to slowing the progression of chronic kidney disease (CKD) (Higdon and Frei 2003). Furthermore, EGCG has been demonstrated to modulate various pathways, including Nrf2/HO‐1, protect mitochondrial function, inhibit fibrosis and abnormal cell proliferation, and alleviate inflammation and oxidative damage associated with CKD (Zhong et al. 2019; Baek et al. 2019; Kanlaya and Thongboonkerd 2019).

Although epidemiological studies suggest that tea consumption may have protective effects against CKD, the specific impact of epigallocatechin gallate (EGCG)—the primary bioactive compound in green tea—remains unclear, and human evidence is sparse (Zhang et al. 2022). Experimental studies have demonstrated that EGCG can mitigate adenine‐induced kidney injury in animal models; however, evidence in humans is limited (Chen and Lin 2015). Furthermore, the dose–response relationship between EGCG intake and CKD risk has yet to reach a consensus, with some studies suggesting a nonlinear association. Some studies indicate that low doses of EGCG improve proteinuria and renal histopathology scores in diabetic nephropathy models, while higher doses, particularly those exceeding 800 mg per day, may lead to adverse effects such as hepatotoxicity (Zhang and Zhang 2018; Parn et al. 2022). This paradox suggests that the influence of EGCG on CKD may involve a complex dose‐dependent relationship, thereby warranting further research to elucidate its safe dosage range and mechanisms of action. Moreover, most existing studies have evaluated tea intake as a whole rather than quantifying EGCG intake. In this study, we estimated EGCG exposure based on individual‐level tea consumption combined with standardized EGCG content data from national and international dietary composition references.

The National Health and Nutrition Examination Survey (NHANES), a large‐scale epidemiological program that is nationally representative, provides detailed dietary data suitable for analyzing the relationship between EGCG intake and kidney function. This study aims to quantify EGCG intake from tea consumption, define CKD using both eGFR and albuminuria indicators according to clinical guidelines, and apply restricted cubic spline (RCS) modeling to examine the potential nonlinear association between EGCG intake and CKD risk. Our findings may help define the safe range of EGCG intake, inform public health recommendations on tea consumption, and provide a basis for further investigation into dietary strategies for CKD prevention.

## 2 Methods

### 2.1 Data Source and CKD Classification Criteria

This study was based on data from the National Health and Nutrition Examination Survey (NHANES) 2009–2018. The initial merged dataset contained information from 49,693 participants. To identify the analytic sample, a stepwise inclusion process was applied. First, participants who had non‐missing tea consumption data from either day of the dietary recall were selected, totaling 9358 individuals. Among them, 7919 had complete data on both estimated glomerular filtration rate (eGFR) and urinary albumin‐to‐creatinine ratio (UACR). After excluding individuals under 18 years of age, 7250 adults remained. Lastly, participants with missing data on covariates were excluded, resulting in a final analytic sample of 5001 adults.

The primary outcome was chronic kidney disease (CKD), defined according to established clinical guidelines as either reduced glomerular filtration rate (eGFR) or elevated albuminuria. Participants were classified as having CKD if they had an eGFR < 60 mL/min/1.73 m^2^ and/or a urine albumin‐to‐creatinine ratio (UACR) ≥ 30 mg/g. Serum creatinine was measured using an enzymatic method and standardized to be traceable to isotope dilution mass spectrometry. Estimated glomerular filtration rate (eGFR) was calculated using the CKD‐EPI 2009 equation (KDIGO 2024):

$$\text{eGFR}=141\times \min{\left(\frac{\kappa }{\mathrm{Scr}},1\right)}^{\alpha }\times \max{\left(\frac{\kappa }{\mathrm{Scr}},1\right)}^{-1.209}\times 0.993^{\mathrm{Age}}\times 1.018$$

where Scr is serum creatinine in mg/dL, *κ* is 0.7 for females and 0.9 for males, and *α* is −0.329 for females and −0.411 for males.

Urine albumin and urine creatinine were measured from spot urine samples. UACR was calculated using the following formula:

$$\text{UACR}\left(\mathrm{mg}/g\right)=\frac{\text{Urine albumin}\left(\mathrm{mg}/\mathrm{dL}\right)}{\text{Urine creatinine}\left(g/\mathrm{dL}\right)}$$

Albuminuria was defined as UACR ≥ 30 mg/g, which corresponds to at least moderately increased albumin excretion (formerly termed microalbuminuria) (Johnson et al. 2012). Given the skewed distribution of UACR, values were log_10_‐transformed when used as a continuous outcome in regression models.

**FIGURE 1:**
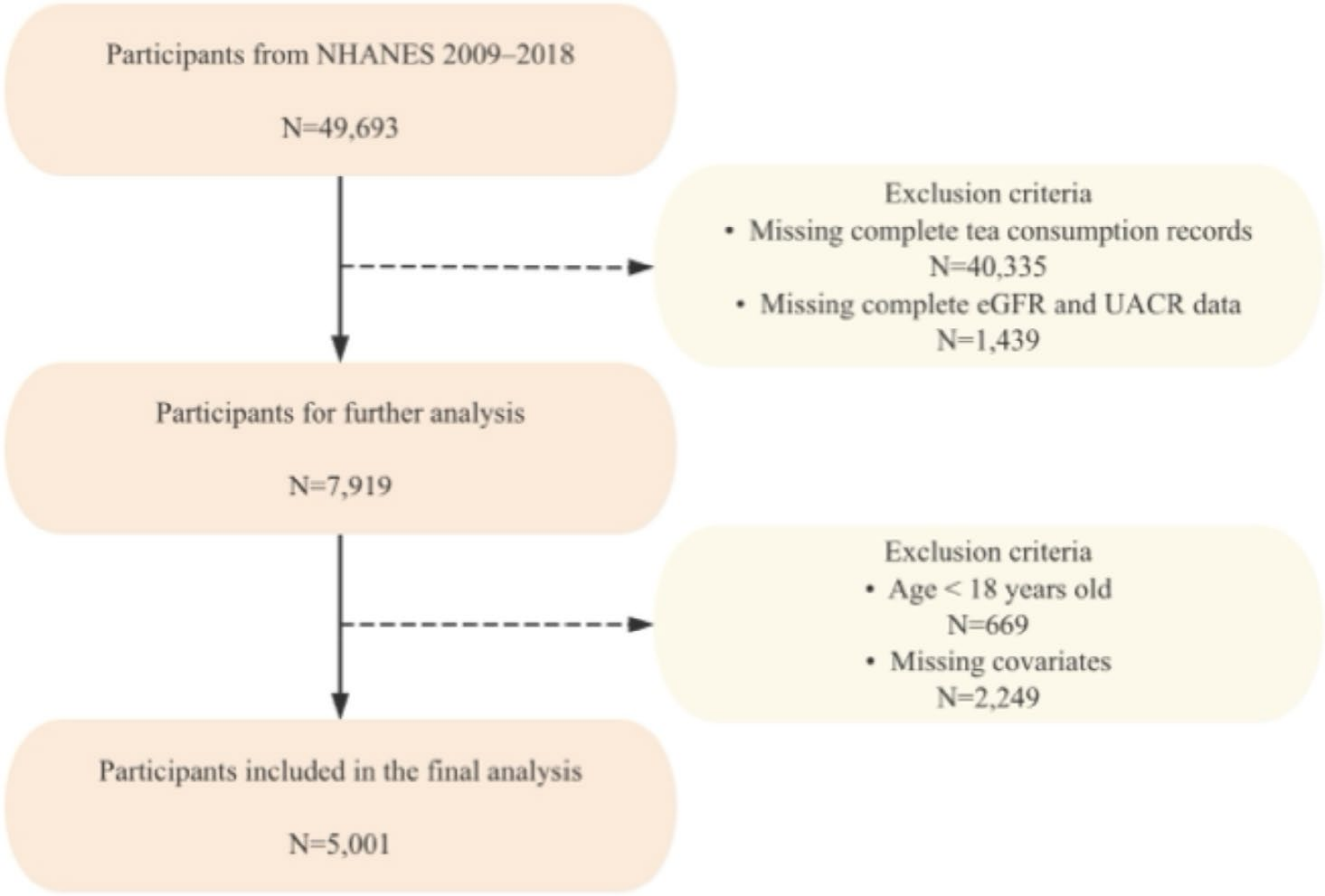
Flowchart of participant inclusion and exclusion.

This study utilized publicly available NHANES data, which had been approved by the National Center for Health Statistics (NCHS) Ethics Review Board. All participants provided informed consent (Centers for Disease Control and Prevention 2024a, 2024b) (Figure 1).

### 2.2 Dietary Data and EGCG Intake Estimation

In this study, the intake of EGCG was estimated based on tea consumption data recorded in the 24‐h dietary recall questionnaires from the National Health and Nutrition Examination Survey (NHANES). The NHANES 24‐h dietary recall data provide detailed information on the daily intake of various foods and beverages, including the amount of each item consumed (in grams). Specifically, the survey documented the type and corresponding amount of tea consumed by each participant. Based on the NHANES dietary intake data, the daily tea consumption for each participant was quantified in grams of brewed tea, which is approximately equivalent to milliliters. To address this limitation, we carefully reviewed existing literature and authoritative sources. Based on data from the European Food Safety Authority (EFSA) and multiple epidemiological studies, we adopted a conservative and widely accepted estimate that green tea beverages contain approximately 0.25 mg of epigallocatechin‐3‐gallate (EGCG) per milliliter (EFSA Panel on Food Additives and Nutrient Sources added to Food (ANS) et al. 2018). Using this value, we estimated each participant's daily EGCG intake (mg/day) by calculating the total volume of tea consumed (from Day 1 and Day 2 recalls) and multiplying it by 0.25 mg/mL. Averaging across the two days of dietary recall helps reduce intra‐individual variability and improves the accuracy of dietary exposure assessment (Knüppel et al. 2019; Bailey 2021).

### 2.3 Covariates

We adjusted for a comprehensive set of demographic, socioeconomic, and lifestyle covariates that are known to influence chronic kidney disease (CKD) risk. Demographic variables included age (continuous), sex (male or female), and race/ethnicity (Non‐Hispanic White, Non‐Hispanic Black, Hispanic, Asian, and Other). Socioeconomic status was assessed using education level (< 9th grade, some high school, high school/GED, some college, and college or above) and the poverty‐income ratio (PIR, continuous). Lifestyle factors included smoking status (current smoker vs. non‐smoker), alcohol consumption (any vs. none in the past 12 months), and physical activity (any vs. none of moderate‐to‐vigorous leisure‐time activity). Body mass index (BMI) was calculated as weight in kilograms divided by height in meters squared (kg/m^2^), and categorized as < 25 (normal or underweight), 25–29.9 (overweight), and ≥ 30 (obese). Diabetes status was defined based on self‐reported physician diagnosis or current use of glucose‐lowering medications. Hypertension was defined as having an average systolic blood pressure of ≥ 130 mmHg, diastolic pressure of ≥ 80 mmHg, self‐reported diagnosis, or current use of antihypertensive medications.

All covariates were derived from the NHANES interview or examination data and coded as categorical or continuous variables as appropriate.

### 2.4 Statistical Analysis

This study utilized complex sampling weights from NHANES data to conduct weighted analyses, ensuring that the results are nationally representative. Descriptive statistics were used to summarize baseline characteristics of participants across CKD status, as defined jointly by eGFR and UACR. Continuous variables were reported as medians with interquartile ranges (Median [IQR]), and categorical variables were expressed as frequencies and percentages (*n* [%]). Group differences were analyzed using the Kruskal–Wallis test for continuous variables and the chi‐squared test for categorical variables. To examine the association between EGCG intake and kidney outcomes, multivariable regression analyses were performed. Logistic regression models were used to assess the relationship between EGCG intake and the risk of CKD, while linear regression models were employed to evaluate the associations with eGFR and UACR levels. All models were adjusted for potential confounders, including age, gender, race/ethnicity, education level, family income‐to‐poverty ratio, body mass index (BMI), smoking status, alcohol consumption, diabetes, hypertension, and physical activity. To investigate potential nonlinear associations between EGCG intake and kidney‐related outcomes, restricted cubic spline (RCS) regression models were applied. Separate models were constructed with EGCG intake as the independent variable and CKD (as a binary outcome), eGFR, and UACR (as continuous outcomes) as dependent variables. The models were adjusted for the same set of covariates as the main regression analyses. To assess the robustness of the observed nonlinear patterns, sensitivity analyses were conducted by varying the number of knots from three to eight. All statistical tests were two‐sided, with a significance level set at p<0.05. Statistical analyses were conducted using R (version 4.2.2).

## 3 Results

### 3.1 Descriptive Statistical Analysis of Study Participants

A total of 5001 participants were included in the analysis, of whom 56.2% were female, and the overall prevalence of CKD was 5.3% (Table 1). Age, sex, race/ethnicity, BMI, socioeconomic status, lifestyle factors, and comorbidities were significantly associated with CKD status. Participants with CKD were more likely to be older, male, Non‐Hispanic Black, and to have diabetes or hypertension (all *p* < 0.001). Median eGFR decreased and UACR increased progressively with age, and males exhibited lower eGFR and higher CKD prevalence than females.

**TABLE 1:** Baseline characteristics of the study population.

Variable	*N*	CKD	Non‐CKD	*p*	eGFR median (IQR)	*p*	UACR median (IQR)	*p*
Age				< 0.001		< 0.001		< 0.001
< 40	1441 (28.8%)	114 (7.9%)	1327 (92.1%)		123.6 [110.3, 134.8]		60 [40.9, 98.6]	
40–60	1825 (36.5%)	101 (5.5%)	1724 (94.5%)		105.9 [92.8, 115.6]		67.9 [46.2, 125]	
≥ 60	1735 (34.7%)	49 (2.8%)	1686 (97.2%)		86.5 [69.1, 96.7]		96.2 [59.7, 209.1]	
Gender				< 0.001		< 0.001		< 0.001
Male	2191 (43.8%)	183 (8.4%)	2008 (91.6%)		91.4 [75.9, 105.8]		62.6 [40.7, 130]	
Female	2810 (56.2%)	81 (2.9%)	2729 (97.1%)	< 0.001	111.4 [96.6, 126.4]		80.4 [54.2, 145.2]	
Race				0.00473		< 0.001		< 0.001
Mexican American	624 (12.5%)	18 (2.9%)	606 (97.1%)		109.4 [94.4, 124.5]		75 [50.9, 154.6]	
Other Hispanic	477 (9.5%)	21 (4.4%)	456 (95.6%)		106.8 [94.5, 117.5]		76.2 [51.2, 143.9]	
Non‐Hispanic White	2146 (42.9%)	117 (5.5%)	2029 (94.5%)		95.4 [80.6, 110.3]		74.4 [46.3, 138.4]	
Non‐Hispanic Black	915 (18.3%)	66 (7.2%)	849 (92.8%)		109.8 [87.7, 128.5]		71.4 [44.3, 147.6]	
Other Race	839 (16.8%)	42 (5%)	797 (95%)		107.7 [92.9, 122.1]		71.7 [47.1, 125.1]	
Education level				0.0349		< 0.001		< 0.001
< 9th grade	369 (7.4%)	8 (2.2%)	361 (97.8%)		97.4 [83.2, 114]		90.9 [56.9, 207.1]	
9–11th grade	545 (10.9%)	29 (5.3%)	516 (94.7%)		104.1 [86.5, 116.9]		73.9 [47.1, 154.6]	
High school graduate	992 (19.8%)	46 (4.6%)	946 (95.4%)		101.2 [84.2, 116]		78.9 [50, 163.4]	
Some college or AA degree	1550 (31%)	79 (5.1%)	1471 (94.9%)		103.3 [87.7, 121]		73.7 [45.5, 137.5]	
College graduate or above	1545 (30.9%)	102 (6.6%)	1443 (93.4%)		103.8 [86.8, 119.3]		66.9 [45.2, 115.7]	
Physical activity				0.0198		0.908		< 0.001
Yes	897 (17.9%)	62 (6.9%)	835 (93.1%)		103 [86.5, 117.4]		65.6 [42.1, 119.4]	
No	4104 (82%)	202 (4.9%)	3902 (95.1%)		102.8 [86.2, 118.8]		75.3 [48.4, 144.4]	
BMI				< 0.001		< 0.001		< 0.001
< 18.5	65 (1.3%)	62 (95.4%)	3 (4.6%)		114.6 [99, 128.9]		119 [67.2, 181.2]	
18.5–25	1408 (28.2%)	1327 (94.2%)	81 (5.8%)		106.8 [89.5, 121.4]		70.4 [46.2, 127]	
> 25	3528 (70.5%)	3348 (94.9%)	180 (5.1%)		101.4 [84.6, 116.8]		74.3 [47.4, 144.2]	
Poverty income ratio				0.0213		0.00152		0.00211
< 1.3	1241 (27%)	1182 (95.2%)	59 (4.8%)		105.1 [88.2, 121.9]		76.4 [47.8, 174]	
1.3–3.5	1729 (37.6%)	1640 (94.9%)	89 (5.1%)		102.5 [84.3, 118.8]		76.9 [48.6, 151]	
> 3.5	1626 (35.4%)	1521 (93.5%)	105 (6.5%)		102 [85.7, 115.9]		66.7 [45.3, 115.4]	
Smoking				0.547		< 0.001		0.00875
Yes	1991 (39.8%)	100 (5%)	1891 (95%)		98.5 [82.9, 112.9]		76.4 [47.9, 156.1]	
No	3010 (60.1%)	164 (5.5%)	2846 (94.5%)		105.9 [88.7, 122.2]		72.2 [46.7, 130.3]	
Drinking				0.0816		0.00217		< 0.001
Yes	3494 (69.9%)	197 (5.6%)	3297 (94.4%)		102.1 [85.9, 117.4]		70.8 [45.4, 133.2]	
No	1507 (30.1%)	67 (4.4%)	1440 (95.6%)		104.8 [87, 120.6]		79.4 [52, 157.5]	
Diabetes				< 0.001		< 0.001		< 0.001
Yes	716 (14.3%)	8 (1.1%)	708 (98.9%)		92.4 [74.4, 106.8]		130 [71.6, 397.9]	
No	4285 (85.6%)	256 (6%)	4029 (94%)		104.6 [88.3, 119.7]		68.4 [45, 121.3]	
Hypertension				< 0.001		< 0.001		< 0.001
Yes	1931 (38.6%)	62 (3.2%)	1869 (96.8%)		92.5 [75.5, 107.7]		93 [57.1, 216.7]	
No	3070 (61.3%)	202 (6.6%)	2868 (93.4%)		109.2 [93.8, 124]		63.6 [43.6, 110.3]	
Tea group				0.0952		0.0317		< 0.001
Low	1667 (33.3%)	72 (4.3%)	1595 (95.7%)		103.7 [87.5, 119.6]		78 [50, 143.4]	
Medium	1667 (33.3%)	98 (5.9%)	1569 (94.1%)		102.7 [85.7, 119.5]		74.1 [45.8, 146.4]	
High	1667 (33.3%)	94 (5.6%)	1573 (94.4%)		102.1 [85.4, 116.2]		68.7 [45.7, 127.8]	
EGCG group				0.0739		0.0539		0.0154
Low	1667 (33.3%)	72 (4.3%)	1595 (95.7%)		103.7 [87.5, 119.6]		78 [50, 143.4]	
Medium	1667 (33.3%)	98 (5.9%)	1569 (94.1%)		102.7 [85.7, 119.5]		74.1 [45.8, 146.4]	
High	1667 (33.3%)	94 (5.6%)	1573 (94.4%)		102.1 [85.4, 116.2]		68.7 [45.7, 127.8]	

BMI showed a graded relationship with kidney function, with underweight participants having the highest eGFR and UACR, and those with BMI ≥ 25 kg/m^2^ having the lowest eGFR (all *p* < 0.001). Lower education level and lower poverty‐income ratio were associated with reduced kidney function, while smoking and drinking were related to eGFR and UACR but not CKD prevalence. Physical activity was associated with lower UACR but not eGFR. In descriptive analysis, tertiles of tea and EGCG intake were not significantly associated with CKD prevalence, although tea intake was related to both eGFR and UACR, and EGCG intake was related to UACR.

### 3.2 Association Between Tea and EGCG Intake and Kidney Function

In logistic models, EGCG intake was not associated with CKD (Table 2): crude OR≈1.03 (95% CI 0.93–1.15) and adjusted OR≈1.09 (95% CI 0.97–1.23), indicating estimates close to the null and little change after adjustment for demographic, lifestyle, and clinical covariates. In contrast, higher EGCG intake was consistently associated with lower eGFR (Table 2): crude *β* ≈ −1.01 mL/min/1.73 m^2^ (95% CI −1.59 to −0.43) and adjusted *β* ≈ −0.86 mL/min/1.73 m^2^ (95% CI −1.23 to −0.49), with only modest attenuation after adjustment, suggesting the association was not explained by measured confounders. For UACR, the regression coefficients were small and not statistically significant in both crude and adjusted models (Table 2)—crude *β* ≈ −13.09 (95% CI −89.26 to 63.08) and adjusted *β* ≈ −22.49 (95% CI −103.87 to 58.90)—indicating no clear relationship between EGCG intake and urinary albumin excretion in this population. All effects scaled per 100 mg/day of EGCG.

**TABLE 2:** Associations of EGCG intake with CKD, eGFR, and UACR in crude and adjusted models.

Outcomes	Model	OR	*β*	95% CI	*p*
CKD	Model 1	1.03		(0.93–1.15)	0.590
	Model 2	1.09		(0.97–1.23)	0.136
eGFR	Model 1		−1.01	(−1.59, −0.43)	0.0006
	Model 2		−0.86	(−1.23, −0.49)	< 0.001
UACR	Model 1		−13.09	(−89.26, 63.08)	0.7362
	Model 2		−22.49	(−103.87, 58.90)	0.5880

### 3.3 Nonlinear Dose–Response Relationships Between EGCG Intake and Kidney Function Indicators: Restricted Cubic Spline Analysis

**FIGURE 2:**
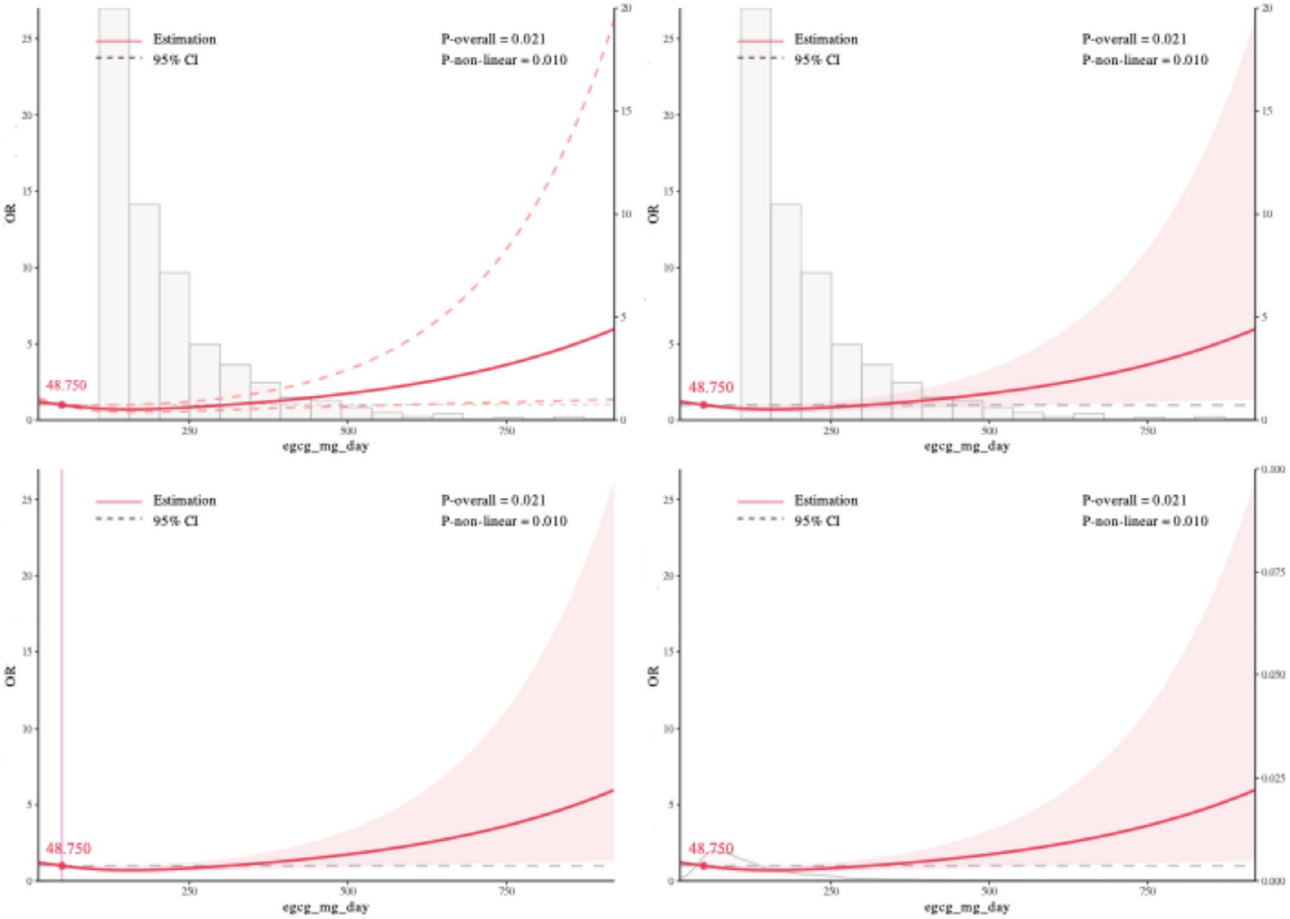
Association of daily EGCG intake with the odds of chronic kidney disease (CKD) using restricted cubic spline analysis.

**FIGURE 3:**
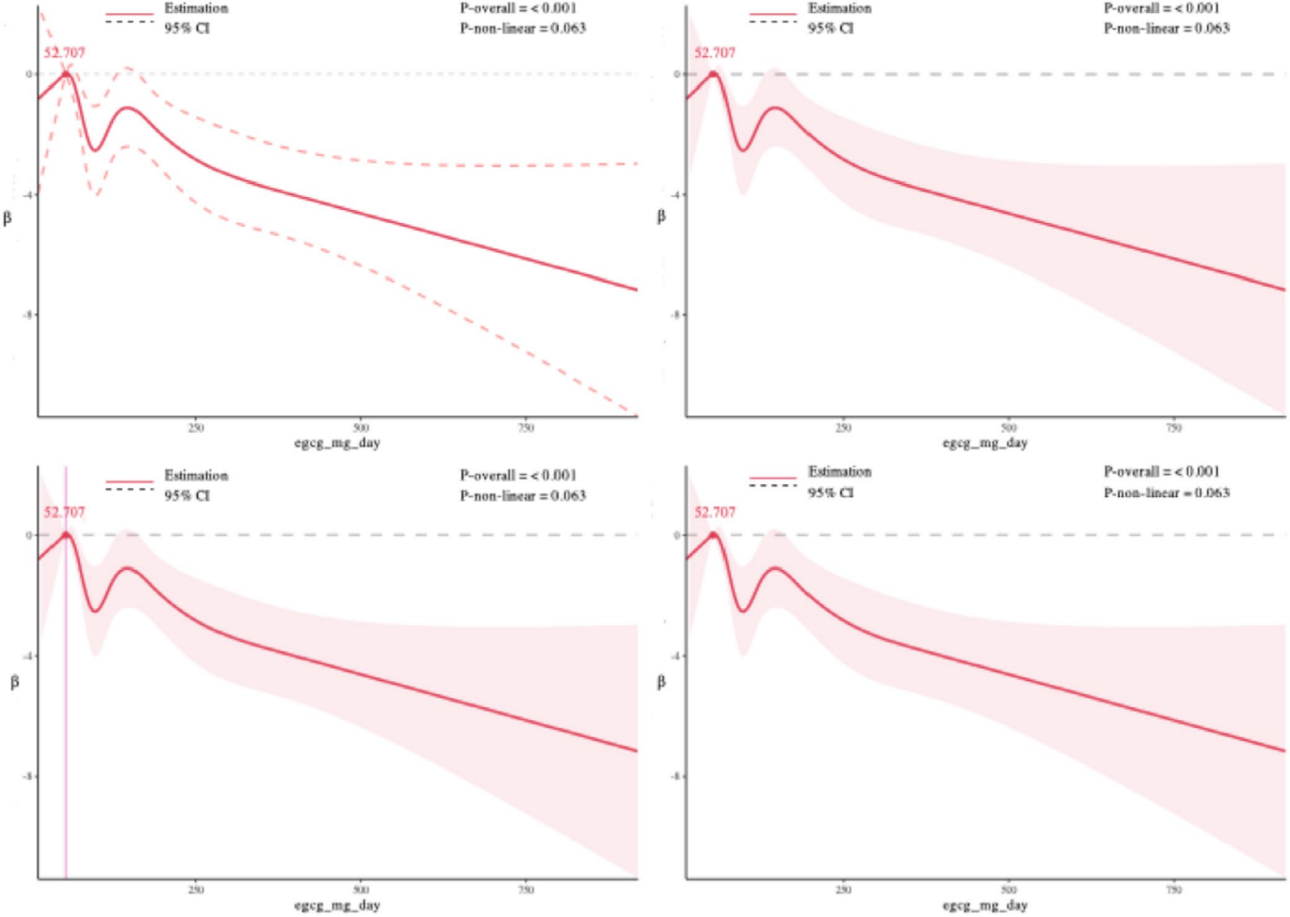
Association of daily EGCG intake with estimated glomerular filtration rate (eGFR) using restricted cubic spline analysis.

**FIGURE 4:**
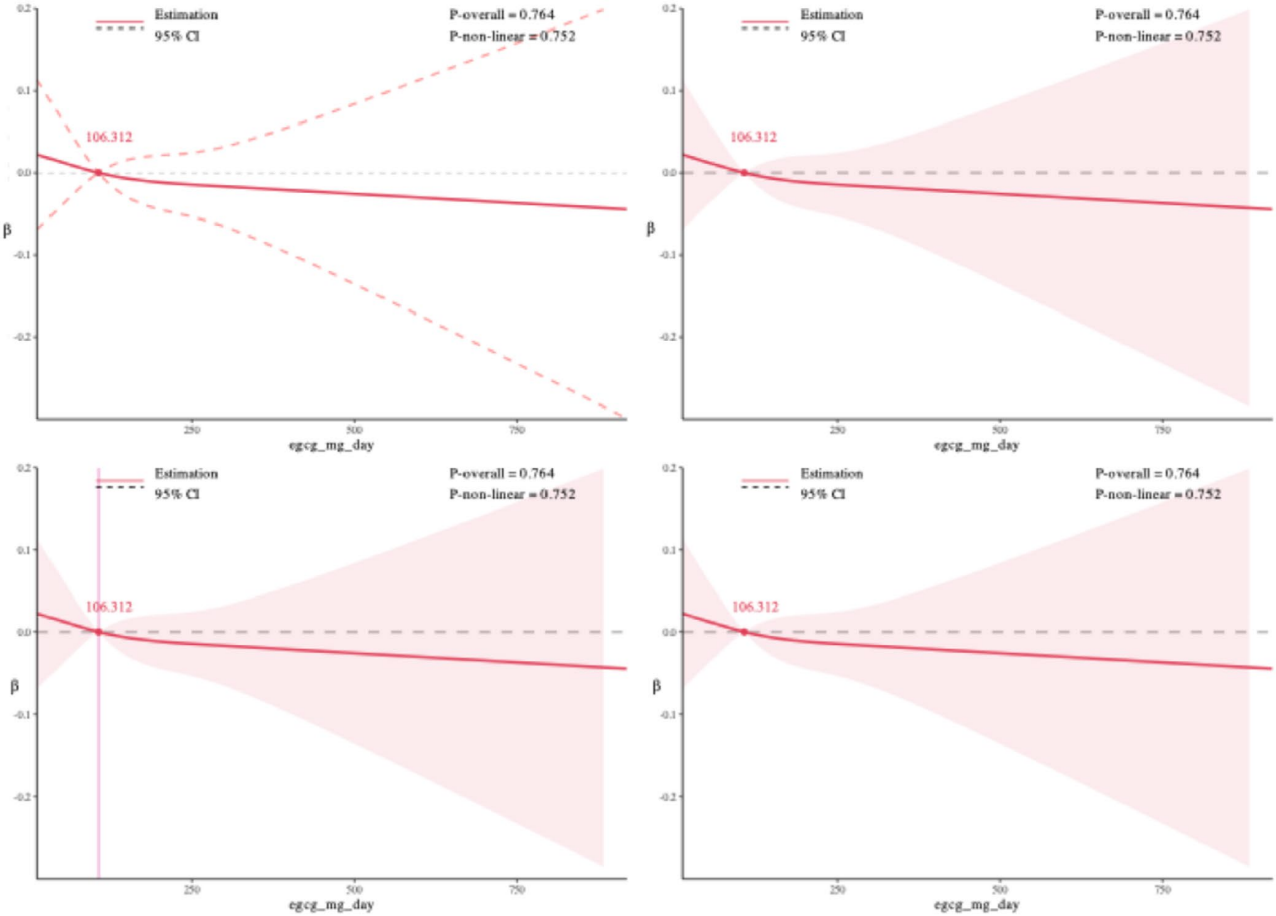
Association of daily EGCG intake with urinary albumin‐creatinine ratio (UACR) using restricted cubic spline analysis.

Restricted cubic spline (RCS) models were used to examine potential nonlinear associations between daily EGCG intake and kidney outcomes, setting 48.750 mg/day as the reference for CKD, 52.707 mg/day for eGFR, and 106.312 mg/day for UACR. For CKD, a significant overall association was observed (*p*‐overall = 0.021), and the nonlinearity test was also significant (*p*‐nonlinear = 0.010). The dose–response curve suggested a nonlinear positive association: CKD risk showed a slight decline at lower EGCG doses, followed by a marked increase at higher intake levels. For eGFR, there was a highly significant overall association (*p*‐overall < 0.001), and the curve displayed an apparent N‐shaped pattern, with an initial increase in eGFR followed by a decrease. However, the test for nonlinearity did not reach statistical significance (*p*‐nonlinear = 0.063), indicating that the observed pattern was not statistically robust. For UACR, neither the overall association (*p*‐overall = 0.764) nor the test for nonlinearity (*p*‐nonlinear = 0.752) was statistically significant, suggesting no evidence of a dose–response relationship between EGCG intake and UACR. Overall, these findings suggest a nonlinear association between EGCG intake and CKD risk, a potential but statistically non‐robust N‐shaped pattern for eGFR, and no meaningful dose–response relationship for UACR (Figures 2, 3, 4).

### 3.4 Sensitivity Analyses

To evaluate the robustness of the dose–response relationships, sensitivity analyses were performed by varying the number of knots in the restricted cubic spline models from 3 to 8. For CKD and eGFR, the shape and statistical significance of the associations remained generally consistent across different knot settings, supporting the stability of the findings. For UACR, the overall trend was similar, but statistical significance for non‐linearity was less consistent, suggesting greater sensitivity to knot selection. Detailed results are provided in Figures S1–S18.

## 4 Discussion

This study utilized data from the National Health and Nutrition Examination Survey (NHANES) to systematically evaluate the dose–response relationship between epigallocatechin gallate (EGCG) intake and the risk of chronic kidney disease (CKD). We also examined tea intake per se and two continuous kidney outcomes—estimated glomerular filtration rate (eGFR) and urine albumin‐to‐creatinine ratio (UACR, including log‐UACR). Exposures were modeled continuously; we fitted conventional linear and logistic regressions and then applied restricted cubic spline (RCS) models, with sensitivity analyses varying the number of knots (3–8). In conventional linear/logistic models, tea or EGCG intake showed no association with CKD. RCS suggested possible curvature, but the non‐linear signal was not robust to knot placement and diminished in sensitivity analyses. For eGFR, higher tea and EGCG intake related to very small, statistically detectable yet clinically trivial decreases; UACR (including log‐UACR) associations were inconsistent and near null across models.

Biologically, modest renal effects of tea catechins are plausible (Bao et al. 2016). EGCG exerts antioxidant and anti‐inflammatory actions, including activation of cytoprotective signaling (e.g., Nrf2/HO‐1) and dampening of NF‐κB–mediated pathways, and it may attenuate profibrotic TGF‐β/Smad signaling and extracellular‐matrix deposition (Thangapandiyan and Miltonprabu 2014; Wang et al. 2015; Liu et al. 2025). Experimental work further suggests benefits on podocyte integrity and endothelial function, with potential improvements in nitric‐oxide bioavailability and intrarenal hemodynamics (Hayashi et al. 2020; Borges et al. 2016; Mohd Sabri et al. 2022). These mechanisms could translate into lower albuminuria or slower GFR decline. However, such effects—if present at habitual dietary intakes—appear small at the population level in our data.

Several features of nutritional epidemiology likely attenuate observable associations. First, EGCG bioavailability is low and highly variable due to extensive phase‐II metabolism and gut‐microbial transformation, creating wide between‐person differences in circulating bioactive that dietary recalls cannot capture (Andreu‐Fernández et al. 2020; Lee et al. 2002; Chow et al. 2005). Second, beverage‐level determinants of catechin dose—tea type, leaf quality, brewing time/temperature, and additions such as milk or sugar—are incompletely measured (Saklar et al. 2015; Koch et al. 2017). Third, 24‐h recalls introduce random and systematic error; using one or two recalls per individual reduces day‐to‐day variation but still yields classical attenuation of diet–disease relations without dedicated measurement‐error correction (Freedman et al. 2017; Kipnis et al. 2002). These epidemiological challenges may help explain the discrepancy between strong preclinical mechanistic evidence and the largely null or minimal associations observed in our population‐based analyses.

Our study has several strengths. We analyzed a large, nationally representative sample; considered complementary kidney endpoints (binary CKD and continuous eGFR/UACR, including log‐UACR); modeled tea and EGCG as continuous exposures to avoid arbitrary categorization; and explicitly examined non‐linearity using RCS with prespecified sensitivity analyses (knots = 3–8), showing that our central conclusions do not hinge on a single spline specification. We also adjusted for a broad set of demographics, socioeconomic, and lifestyle covariates that are relevant to CKD risk.

Limitations should temper interpretation. The cross‐sectional design precludes causal inference and raises the possibility of reverse causation. Exposure misclassification is likely: converting tea volume to EGCG with a uniform factor cannot reflect variability from tea variety, preparation, and co‐injectants that influence extraction and absorption. Non‐linearity analyses at the lowest and highest intakes remain underpowered due to sparse data in the tails, making shape estimates sensitive to knot placement; our sensitivity checks accordingly favored conservative inference.

In sum, across complementary outcomes and modeling strategies, we found no stable, clinically meaningful association between tea or EGCG intake and CKD, minute population‐level decrements in eGFR with higher intake, and largely null results for UACR. Within the range of tea consumption observed in the US population, these findings do not support modifying tea or EGCG intake specifically to improve kidney outcomes. Future research should prioritize prospective cohorts with repeated diet assessment, objective biomarkers of catechin exposure (including microbial metabolites), explicit differentiation of tea types and preparation methods, and adequately powered tests of non‐linearity across a wider intake spectrum—ideally complemented by causal‐inference approaches or randomized trials to determine whether any small effects are real and clinically relevant.

## 5 Conclusion

Using nationally representative NHANES data with both conventional regression and restricted cubic splines, we found no stable association between tea or EGCG intake and CKD. For eGFR, higher intake related to very small, clinically trivial decreases; associations with UACR were largely null. Spline analyses did not reveal a consistent non‐linear pattern across knot choices. Overall, these results do not support modifying tea or EGCG intake specifically to improve kidney outcomes in the general population.

## Author Contributions

Xuetong Tang conceptualized the study, collected and processed the data, performed the statistical analyses, and drafted the manuscript. Jiankui Guo and Yuan Liu contributed to the revision and proofreading of the manuscript. Yuan Liu and Wen Hu supervised and guided the research process. All authors read and approved the final version of the manuscript.

## Conflicts of Interest

The authors declare no conflicts of interest.

## Supporting information


**Figure S1:** fsn370997‐sup‐0001‐FigureS1‐S18.docx. **Figure S1–S6** Restricted cubic spline (RCS) for the association between EGCG intake and CKD with 3–8 knots, respectively. The solid curve shows the adjusted odds ratio (OR) relative to the reference exposure; shaded areas denote 95% confidence intervals. The vertical dashed line marks the reference exposure. Models adjust for age, sex, race/ethnicity, education, poverty‐income ratio, BMI, smoking, alcohol use, diabetes, hypertension, and leisure‐time physical activity. **Figure S7–S12**. RCS for the association between EGCG intake and eGFR with 3–8 knots, respectively. The solid curve shows the adjusted spline‐predicted β relative to the reference exposure; shaded areas denote 95% confidence intervals. Covariate adjustment **Figure S13–S18**. RCS for the association between EGCG intake and log‐UACR with 3–8 knots, respectively. The solid curve shows the adjusted spline‐predicted β relative to the reference exposure; shaded areas denote 95% confidence intervals. Covariate adjustment as in Figure S1.

## Data Availability

This study utilized data from the National Health and Nutrition Examination Survey (NHANES), covering the 2009–2018 cycles. The NHANES datasets are publicly available and can be accessed at https://www.cdc.gov/nchs/nhanes/index.htm. Detailed variable documentation, data dictionaries, and survey methodologies are provided on the NHANES website. All analyses in this study were conducted in accordance with NHANES guidelines and ethical standards for secondary data use.
