# Supplementary material for: Effect of EGCG Intake on Kidney Function Chronic Kidney Disease: A Nonlinear Dose–Response Relationship and Health Impact Evidence From US Adults
Source: Food Sci Nutr. 2025 Oct 29;13(11):e70997. doi: 10.1002/fsn3.70997 (PMC12569527; doi:10.1002/fsn3.70997)
Supplement: Supplementary file 1 — Figure S1: fsn370997‐sup‐0001‐FigureS1‐S18.docx. Figure S1–S6 Restricted cubic spline (RCS) for the association between EGCG intake and CKD with 3–8 knots, respectively. The solid curve shows the adjusted odds ratio (OR) relative to the reference exposure; shaded areas denote 95% confidence intervals. The vertical dashed line marks the reference exposure. Models adjust for age, sex, race/ethnicity, education, poverty‐income ratio, BMI, smoking, alcohol use, diabetes, hypertension, and leisure‐time physical activity. Figure S7–S12. RCS for the association between EGCG intake and eGFR with 3–8 knots, respectively. The solid curve shows the adjusted spline‐predicted β relative to the reference exposure; shaded areas denote 95% confidence intervals. Covariate adjustment Figure S13–S18. RCS for the association between EGCG intake and log‐UACR with 3–8 knots, respectively. The solid curve shows the adjusted spline‐predicted β relative to the reference exposure; shaded areas denote 95% confidence intervals. Covariate adjustment as in Figure S1. [file FSN3-13-e70997-s001.docx]

**Supplement Information**

**Effect of EGCG Intake on Kidney Function Chronic Kidney Disease: A Nonlinear Dose-Response Relationship and Health Impact Evidence from US Adults**

This file include：

1. **Supplementary figures**

Figures S1 to S18

**Figure S1–S6**. Restricted cubic spline (RCS) for the association between EGCG intake and CKD with 3–8 knots, respectively. The solid curve shows the adjusted odds ratio (OR) relative to the reference exposure; shaded areas denote 95% confidence intervals. The vertical dashed line marks the reference exposure. Models adjust for age, sex, race/ethnicity, education, poverty-income ratio, BMI, smoking, alcohol use, diabetes, hypertension, and leisure-time physical activity.

Figure S1

Figure S2

Figure S3

Figure S4

Figure S5

Figure S6

**Figure S7–S12**. RCS for the association between EGCG intake and eGFR with 3–8 knots, respectively. The solid curve shows the adjusted spline-predicted β relative to the reference exposure; shaded areas denote 95% confidence intervals. Covariate adjustment as in Figure S1.

Figure S7

Figure S8

Figure S9

Figure S10

Figure S11

Figure S12

**Figure S13–S18.** RCS for the association between EGCG intake and log-UACR with 3–8 knots, respectively. The solid curve shows the adjusted spline-predicted β relative to the reference exposure; shaded areas denote 95% confidence intervals. Covariate adjustment as in Figure S1.

Figure S13

Figure S14

Figure S15

Figure S16

Figure S17

Figure S18
